# Tetra­kis(6-methyl-2,2′-bipyridine)-1κ^2^ *N*,*N*′;2κ^2^ *N*,*N*′;3κ^2^ *N*,*N*′;4κ^2^ *N*,*N*′-tetra-μ-nitrato-1:2κ^2^ *O*:*O*′;2:3κ^3^ *O*:*O*′,*O*′′;2:3κ^3^ *O*,*O*′:*O*′′;3:4κ^2^ *O*:*O*′-tetra­nitrato-1κ^4^ *O*,*O*′;4κ^2^ *O*,*O*′-tetra­lead(II)

**DOI:** 10.1107/S160053680903459X

**Published:** 2009-09-05

**Authors:** Roya Ahmadi, Khadijeh Kalateh, Robabeh Alizadeh, Zeinab Khoshtarkib, Vahid Amani

**Affiliations:** a Islamic Azad University, Shahr-e-Rey Branch, Tehran, Iran; b Damghan University of Basic Sciences, School of Chemistry, Damghan, Iran

## Abstract

In the  tetranuclear centrosymmetric title compound, [Pb_4_(NO_3_)_8_(C_11_H_10_N_2_)_4_], irregular PbN_2_O_5_ and PbN_2_O_4_ coordination polyhedra occur. The hepta­coordinated lead(II) ion is bonded to two bidentate and one monodentate nitrate ion and one bidentate 6-methyl-2,2′-bipyridine (mbpy) ligand. The six-coordinate lead(II) ion is bonded to one bidentate and two monodentate nitrate anions and one mbpy ligand. In the crystal, bridging nitrate anions lead to infinite chains propagating in [111]. A number of C—H⋯O hydrogen bonds may stabilize the structure.

## Related literature

For different metal complexes of 6-methyl-2,2′-bipyridine, see: Ahmadi, Kalateh *et al.* (2008 ▶); Ahmadi, Ebadi *et al.* (2008 ▶); Newkome *et al.* (1982 ▶); Onggo *et al.* (1990 ▶, 2005 ▶).
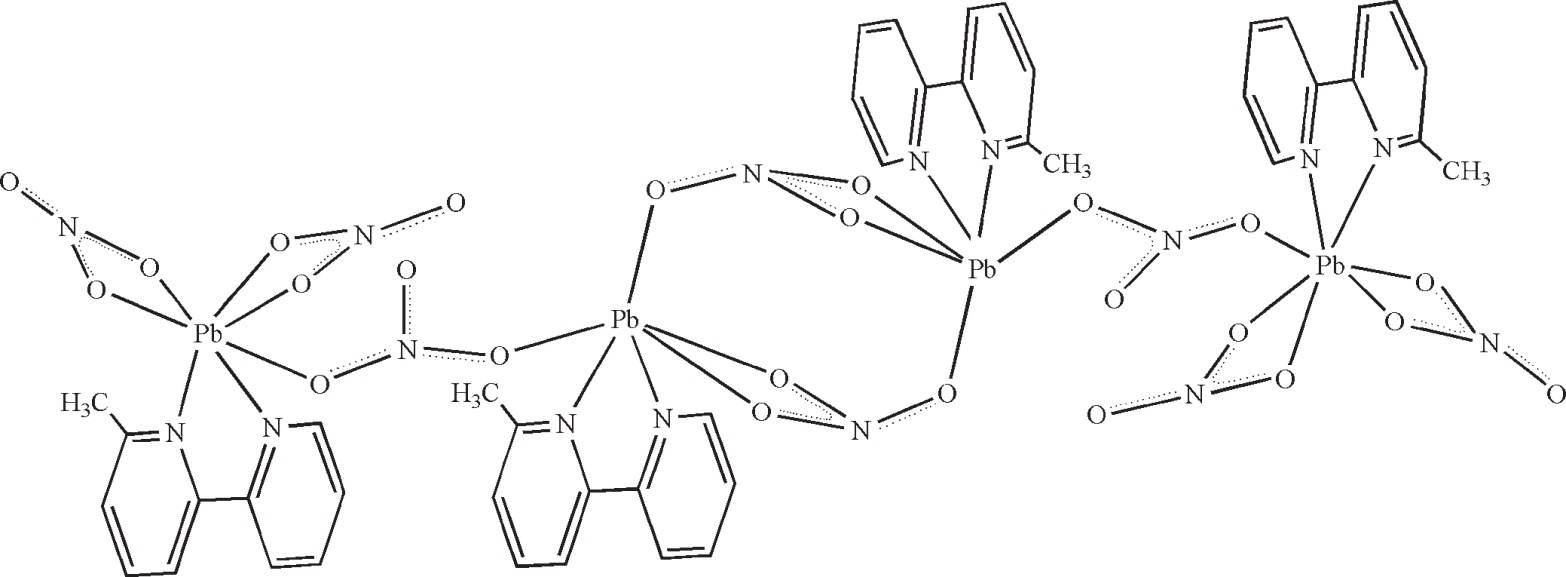


## Experimental

### 

#### Crystal data

[Pb_4_(NO_3_)_8_(C_11_H_10_N_2_)_4_]
*M* *_r_* = 1002.84
Triclinic, 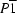

*a* = 11.093 (2) Å
*b* = 11.266 (2) Å
*c* = 12.642 (3) Å
α = 109.25 (3)°
β = 95.43 (3)°
γ = 105.62 (3)°
*V* = 1407.0 (7) Å^3^
*Z* = 2
Mo *K*α radiation
μ = 12.03 mm^−1^
*T* = 298 K
0.40 × 0.30 × 0.25 mm

#### Data collection

Bruker SMART CCD diffractometer
Absorption correction: numerical shape of crystal determined optically (*X-SHAPE* and *X-RED32*; Stoe & Cie, 2005 ▶) *T*_min_ = 0.021, *T*_max_ = 0.052
16752 measured reflections
7561 independent reflections
6277 reflections with *I* > 2σ(*I*)
*R*_int_ = 0.089

#### Refinement

*R*[*F* ^2^ > 2σ(*F* ^2^)] = 0.049
*wR*(*F* ^2^) = 0.137
*S* = 1.10
7561 reflections
397 parameters
H-atom parameters constrained
Δρ_max_ = 2.18 e Å^−3^
Δρ_min_ = −2.37 e Å^−3^

### 

Data collection: *SMART* (Bruker, 1998 ▶); cell refinement: *SAINT* (Bruker, 1998 ▶); data reduction: *SAINT*; program(s) used to solve structure: *SHELXTL* (Sheldrick, 2008 ▶); program(s) used to refine structure: *SHELXTL*; molecular graphics: *ORTEP-3* (Farrugia, 1997 ▶); software used to prepare material for publication: *WinGX* (Farrugia, 1999 ▶).

## Supplementary Material

Crystal structure: contains datablocks I, global. DOI: 10.1107/S160053680903459X/hb5028sup1.cif

Structure factors: contains datablocks I. DOI: 10.1107/S160053680903459X/hb5028Isup2.hkl

Additional supplementary materials:  crystallographic information; 3D view; checkCIF report

## Figures and Tables

**Table 1:** Selected bond lengths (Å)

Pb1—O1	2.566 (8)
Pb1—O2	2.609 (8)
Pb1—O4	2.851 (8)
Pb1—O5	2.675 (9)
Pb1—O8	2.693 (8)
Pb1—N1	2.618 (8)
Pb1—N2	2.528 (8)
Pb2—O9	2.624 (7)
Pb2—O10	2.763 (11)
Pb2—O11	2.629 (8)
Pb2—N6	2.470 (7)
Pb2—N7	2.422 (8)
Pb2—O12^i^	2.910 (9)

Symmetry code: (i) 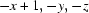.

**Table 2:** Hydrogen-bond geometry (Å, °)

*D*—H⋯*A*	*D*—H	H⋯*A*	*D*⋯*A*	*D*—H⋯*A*
C1—H1*A*⋯O4^ii^	0.96	2.55	3.378 (14)	144
C3—H3⋯O10^iii^	0.93	2.50	3.368 (17)	155
C10—H10⋯O6^iv^	0.93	2.47	3.349 (17)	157
C12—H12*A*⋯O10	0.96	2.48	3.299 (15)	143
C12—H12*B*⋯O7	0.96	2.49	3.412 (17)	160
C12—H12*C*⋯O8^iii^	0.96	2.59	3.516 (14)	162
C19—H19⋯O9^v^	0.93	2.45	3.253 (13)	145
C21—H21⋯O1^iv^	0.93	2.42	3.212 (15)	143
C22—H22⋯O12^i^	0.93	2.53	3.268 (15)	137

Symmetry codes: (i) 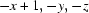; (ii) 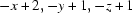; (iii) 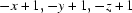; (iv) 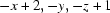; (v) 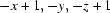.

**Fig. 1.**
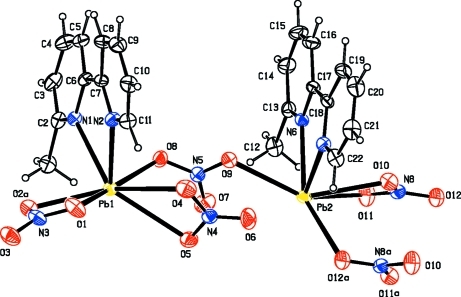
The molecular structure of (I). Displacement ellipsoids are drawn at the 30% probability level. [Symmetry codes: (a) -x,-y,-z]

## supplementary crystallographic information

### Comment

6-Methyl-2,2'-bipyridine (6-mbipy) is a good bidentate ligand, and numerous complexes with 6-mbipy have been prepared, such as that of zinc (Ahmadi, Kalateh *et al.*, 2008), mercury (Ahmadi, Ebadi *et al.*, 2008), palladium (Newkome *et al.*, 1982), ruthenium (Onggo, Scudder *et al.*, 2005) and iron (Onggo, Hook *et al.*, 1990). We report herein the synthesis and crystal structure of the title compound (I).

The asymmetric unit of this tetrameric compound, (I), (Fig. 1), contains two different hepta and hexa-coordinated Pb^II^ centers that the metallic centers site in distorted environment by five oxygen atoms from three nitrate and two nitrogen atom from one 6-methyl-2,2'-bipyridine and distorted octahedral environment by four oxygen atoms from three nitrate and two nitrogen atom from one 6-methyl-2,2'-bipyridine respectively. The Pb—O and Pb—N bond lengths and angles are collected in Table 1.

In the crystal structure, intermolecular C—H···O hydrogen bonds (Table 2) may stabilize the structure.

### Experimental

6-Methyl-2,2'-bipyridine (0.14 g, 0.79 mmol, 0.13 ml) in 5 ml me thanol was added to a solution of Pb(NO_3_)_2_ (0.26 g, 0.79 mmol) in methanol (10 ml) and the resulting colorless solution was stirred at 313 K for 30 min. This solution was left to evaporate slowly at room temperature. After one week, colorless blocks of (I) were isolated (yield 0.29 g, 73.2%).

### Refinement

All H atoms were positioned geometrically, with C—H = 0.93Å and refined as riding with *U*_iso_(H)=1.2*U*_eq_.

### Crystal data

**Table:**

[Pb_4_(NO_3_)_8_(C_11_H_10_N_2_)_4_]	*Z* = 2
*M**_r_* = 1002.84	*F*(000) = 936
Triclinic, *P*1	*D*_x_ = 2.367 Mg m^−^^3^
Hall symbol: -P 1	Mo *K*α radiation, λ = 0.71073 Å
*a* = 11.093 (2) Å	Cell parameters from 1311 reflections
*b* = 11.266 (2) Å	θ = 1.7–29.2°
*c* = 12.642 (3) Å	µ = 12.03 mm^−^^1^
α = 109.25 (3)°	*T* = 298 K
β = 95.43 (3)°	Block, colorless
γ = 105.62 (3)°	0.40 × 0.30 × 0.25 mm
*V* = 1407.0 (7) Å^3^	

### Data collection

**Table:**

Bruker SMART CCD diffractometer	7561 independent reflections
Radiation source: fine-focus sealed tube	6277 reflections with *I* > 2σ(*I*)
graphite	*R*_int_ = 0.089
ω scans	θ_max_ = 29.2°, θ_min_ = 1.7°
Absorption correction: numerical shape of crystal determined optically (program? reference?)	*h* = −15→15
*T*_min_ = 0.021, *T*_max_ = 0.052	*k* = −15→14
16752 measured reflections	*l* = −17→17

### Refinement

**Table:**

Refinement on *F*^2^	Primary atom site location: structure-invariant direct methods
Least-squares matrix: full	Secondary atom site location: difference Fourier map
*R*[*F*^2^ > 2σ(*F*^2^)] = 0.049	Hydrogen site location: inferred from neighbouring sites
*wR*(*F*^2^) = 0.137	H-atom parameters constrained
*S* = 1.10	*w* = 1/[σ^2^(*F*_o_^2^) + (0.0636*P*)^2^ + 3.7094*P*] where *P* = (*F*_o_^2^ + 2*F*_c_^2^)/3
7561 reflections	(Δ/σ)_max_ = 0.011
397 parameters	Δρ_max_ = 2.18 e Å^−^^3^
0 restraints	Δρ_min_ = −2.37 e Å^−^^3^

### Special details

**Table:**

Geometry. All e.s.d.'s (except the e.s.d. in the dihedral angle between two l.s. planes) are estimated using the full covariance matrix. The cell e.s.d.'s are taken into account individually in the estimation of e.s.d.'s in distances, angles and torsion angles; correlations between e.s.d.'s in cell parameters are only used when they are defined by crystal symmetry. An approximate (isotropic) treatment of cell e.s.d.'s is used for estimating e.s.d.'s involving l.s. planes.
Refinement. Refinement of *F*^2^ against ALL reflections. The weighted *R*-factor *wR* and goodness of fit *S* are based on *F*^2^, conventional *R*-factors *R* are based on *F*, with *F* set to zero for negative *F*^2^. The threshold expression of *F*^2^ > σ(*F*^2^) is used only for calculating *R*-factors(gt) *etc*. and is not relevant to the choice of reflections for refinement. *R*-factors based on *F*^2^ are statistically about twice as large as those based on *F*, and *R*- factors based on ALL data will be even larger.

### Fractional atomic coordinates and isotropic or equivalent isotropic displacement parameters (Å^2^)

**Table:**

	*x*	*y*	*z*	*U*_iso_*/*U*_eq_	
C1	0.9335 (12)	0.7297 (9)	0.8235 (9)	0.062 (3)	
H1A	0.9208	0.6913	0.7416	0.074*	
H1B	1.0234	0.7629	0.8558	0.074*	
H1C	0.8983	0.8013	0.8446	0.074*	
C2	0.8691 (9)	0.6277 (9)	0.8670 (7)	0.0478 (18)	
C3	0.7749 (12)	0.6393 (12)	0.9294 (9)	0.066 (3)	
H3	0.7515	0.7159	0.9485	0.079*	
C4	0.7160 (12)	0.5403 (12)	0.9634 (10)	0.067 (3)	
H4	0.6503	0.5476	1.0032	0.080*	
C5	0.7530 (10)	0.4297 (10)	0.9393 (8)	0.053 (2)	
H5	0.7142	0.3619	0.9636	0.063*	
C6	0.8491 (8)	0.4204 (8)	0.8785 (7)	0.0412 (15)	
C7	0.8970 (8)	0.3056 (8)	0.8500 (7)	0.0424 (16)	
C8	0.8691 (9)	0.2158 (9)	0.9059 (9)	0.052 (2)	
H8	0.8187	0.2264	0.9609	0.063*	
C9	0.9175 (12)	0.1117 (9)	0.8781 (10)	0.062 (3)	
H9	0.8987	0.0501	0.9132	0.074*	
C10	0.9918 (11)	0.0992 (10)	0.8002 (10)	0.060 (2)	
H10	1.0288	0.0319	0.7834	0.072*	
C11	1.0127 (10)	0.1894 (9)	0.7449 (9)	0.053 (2)	
H11	1.0577	0.1762	0.6859	0.063*	
C12	0.3946 (11)	0.3441 (9)	0.3475 (10)	0.062 (3)	
H12A	0.3814	0.3192	0.2659	0.075*	
H12B	0.4834	0.3913	0.3805	0.075*	
H12C	0.3446	0.4000	0.3778	0.075*	
C13	0.3544 (9)	0.2208 (9)	0.3761 (8)	0.0505 (19)	
C14	0.2605 (10)	0.2030 (13)	0.4403 (10)	0.068 (3)	
H14	0.2193	0.2660	0.4646	0.081*	
C15	0.2295 (11)	0.0929 (14)	0.4671 (10)	0.075 (3)	
H15	0.1664	0.0798	0.5094	0.091*	
C16	0.2915 (9)	0.0006 (12)	0.4318 (9)	0.062 (3)	
H16	0.2723	−0.0745	0.4507	0.074*	
C17	0.3841 (7)	0.0227 (8)	0.3667 (7)	0.0425 (16)	
C18	0.4550 (8)	−0.0716 (8)	0.3243 (7)	0.0442 (17)	
C19	0.4256 (11)	−0.1938 (10)	0.3387 (9)	0.063 (3)	
H19	0.3604	−0.2185	0.3759	0.076*	
C20	0.4965 (13)	−0.2769 (10)	0.2960 (11)	0.072 (3)	
H20	0.4774	−0.3589	0.3036	0.087*	
C21	0.5926 (12)	−0.2405 (10)	0.2438 (11)	0.067 (3)	
H21	0.6405	−0.2959	0.2160	0.081*	
C22	0.6182 (10)	−0.1186 (10)	0.2325 (10)	0.059 (2)	
H22	0.6836	−0.0931	0.1956	0.071*	
N1	0.9053 (7)	0.5176 (7)	0.8420 (6)	0.0411 (13)	
N2	0.9713 (8)	0.2921 (7)	0.7728 (6)	0.0477 (16)	
N3	1.2895 (7)	0.5378 (9)	0.7956 (8)	0.057 (2)	
N4	0.9322 (8)	0.2233 (8)	0.4204 (7)	0.0519 (17)	
N5	0.7285 (7)	0.3733 (8)	0.4898 (6)	0.0469 (15)	
N6	0.4148 (6)	0.1316 (7)	0.3391 (6)	0.0410 (13)	
N7	0.5523 (7)	−0.0368 (7)	0.2727 (6)	0.0443 (14)	
N8	0.3510 (8)	0.0199 (8)	0.0272 (7)	0.0505 (16)	
O1	1.2315 (7)	0.4180 (7)	0.7307 (8)	0.071 (2)	
O2	1.2252 (7)	0.6164 (7)	0.8206 (7)	0.0591 (16)	
O3	1.4048 (7)	0.5766 (10)	0.8320 (9)	0.083 (3)	
O4	0.9642 (9)	0.3404 (8)	0.4264 (7)	0.068 (2)	
O5	0.9608 (9)	0.2066 (8)	0.5144 (8)	0.076 (2)	
O6	0.8789 (12)	0.1308 (10)	0.3342 (9)	0.110 (4)	
O7	0.7188 (10)	0.4369 (9)	0.4276 (8)	0.082 (3)	
O8	0.7761 (7)	0.4279 (7)	0.5941 (6)	0.0578 (16)	
O9	0.6873 (7)	0.2468 (7)	0.4469 (6)	0.0612 (17)	
O10	0.3682 (10)	0.1365 (8)	0.0852 (8)	0.077 (3)	
O11	0.4108 (7)	−0.0399 (7)	0.0670 (7)	0.0614 (17)	
O12	0.2747 (8)	−0.0373 (8)	−0.0665 (7)	0.070 (2)	
Pb1	1.02005 (3)	0.45241 (3)	0.67017 (2)	0.04043 (9)	
Pb2	0.58525 (3)	0.16179 (3)	0.22876 (3)	0.04202 (10)	

### Atomic displacement parameters (Å^2^)

**Table:**

	*U*^11^	*U*^22^	*U*^33^	*U*^12^	*U*^13^	*U*^23^
C1	0.087 (7)	0.044 (5)	0.063 (6)	0.030 (5)	0.012 (5)	0.024 (4)
C2	0.057 (5)	0.046 (4)	0.046 (4)	0.030 (4)	0.008 (4)	0.014 (3)
C3	0.086 (8)	0.074 (7)	0.052 (5)	0.049 (6)	0.018 (5)	0.021 (5)
C4	0.076 (7)	0.081 (7)	0.065 (6)	0.048 (6)	0.041 (5)	0.028 (5)
C5	0.057 (5)	0.064 (5)	0.048 (4)	0.022 (4)	0.017 (4)	0.029 (4)
C6	0.045 (4)	0.048 (4)	0.038 (3)	0.021 (3)	0.009 (3)	0.020 (3)
C7	0.047 (4)	0.040 (4)	0.042 (4)	0.012 (3)	0.011 (3)	0.019 (3)
C8	0.053 (5)	0.050 (5)	0.067 (5)	0.016 (4)	0.021 (4)	0.036 (4)
C9	0.079 (7)	0.043 (4)	0.073 (6)	0.019 (5)	0.020 (5)	0.032 (5)
C10	0.070 (6)	0.043 (4)	0.075 (6)	0.026 (4)	0.017 (5)	0.026 (4)
C11	0.059 (5)	0.048 (5)	0.061 (5)	0.028 (4)	0.020 (4)	0.022 (4)
C12	0.068 (6)	0.045 (5)	0.078 (7)	0.035 (4)	0.015 (5)	0.013 (4)
C13	0.048 (5)	0.050 (5)	0.049 (4)	0.019 (4)	0.008 (4)	0.010 (4)
C14	0.052 (5)	0.086 (8)	0.067 (6)	0.031 (5)	0.027 (5)	0.019 (6)
C15	0.056 (6)	0.112 (10)	0.064 (6)	0.025 (6)	0.036 (5)	0.035 (6)
C16	0.044 (5)	0.080 (7)	0.064 (6)	0.009 (5)	0.018 (4)	0.038 (5)
C17	0.034 (3)	0.048 (4)	0.044 (4)	0.007 (3)	0.008 (3)	0.021 (3)
C18	0.040 (4)	0.039 (4)	0.048 (4)	0.003 (3)	0.004 (3)	0.019 (3)
C19	0.070 (6)	0.053 (5)	0.066 (6)	0.002 (5)	0.007 (5)	0.037 (5)
C20	0.093 (8)	0.044 (5)	0.082 (7)	0.019 (5)	−0.008 (6)	0.036 (5)
C21	0.083 (8)	0.050 (5)	0.080 (7)	0.039 (5)	0.005 (6)	0.027 (5)
C22	0.053 (5)	0.052 (5)	0.075 (6)	0.026 (4)	0.006 (5)	0.022 (5)
N1	0.046 (4)	0.040 (3)	0.043 (3)	0.022 (3)	0.010 (3)	0.016 (3)
N2	0.060 (4)	0.043 (4)	0.045 (4)	0.020 (3)	0.015 (3)	0.018 (3)
N3	0.038 (4)	0.064 (5)	0.081 (6)	0.012 (3)	0.004 (4)	0.047 (5)
N4	0.057 (4)	0.054 (4)	0.055 (4)	0.019 (3)	0.021 (3)	0.017 (3)
N5	0.038 (3)	0.056 (4)	0.049 (4)	0.020 (3)	0.007 (3)	0.019 (3)
N6	0.035 (3)	0.041 (3)	0.045 (3)	0.012 (3)	0.006 (3)	0.014 (3)
N7	0.052 (4)	0.036 (3)	0.047 (4)	0.017 (3)	0.011 (3)	0.016 (3)
N8	0.051 (4)	0.053 (4)	0.050 (4)	0.018 (3)	0.015 (3)	0.020 (3)
O1	0.054 (4)	0.053 (4)	0.118 (7)	0.021 (3)	0.016 (4)	0.045 (4)
O2	0.054 (4)	0.053 (4)	0.076 (5)	0.024 (3)	0.017 (3)	0.025 (3)
O3	0.043 (4)	0.096 (6)	0.111 (7)	0.012 (4)	0.003 (4)	0.053 (6)
O4	0.088 (6)	0.058 (4)	0.062 (4)	0.015 (4)	0.019 (4)	0.032 (3)
O5	0.086 (6)	0.066 (5)	0.091 (6)	0.027 (4)	0.019 (5)	0.047 (4)
O6	0.121 (9)	0.070 (6)	0.086 (7)	0.011 (6)	0.025 (6)	−0.023 (5)
O7	0.104 (7)	0.089 (6)	0.081 (6)	0.051 (5)	0.020 (5)	0.051 (5)
O8	0.052 (4)	0.065 (4)	0.046 (3)	0.019 (3)	0.011 (3)	0.007 (3)
O9	0.056 (4)	0.063 (4)	0.058 (4)	0.011 (3)	0.009 (3)	0.022 (3)
O10	0.089 (7)	0.059 (5)	0.078 (5)	0.040 (5)	−0.004 (5)	0.023 (4)
O11	0.057 (4)	0.062 (4)	0.075 (5)	0.025 (3)	0.009 (3)	0.033 (4)
O12	0.062 (4)	0.078 (5)	0.059 (4)	0.015 (4)	−0.002 (3)	0.021 (4)
Pb1	0.04429 (17)	0.04041 (16)	0.04192 (15)	0.01671 (12)	0.01061 (11)	0.01886 (12)
Pb2	0.04585 (17)	0.03412 (15)	0.04792 (17)	0.01031 (11)	0.01553 (12)	0.01783 (12)

### Geometric parameters (Å, °)

**Table:**

Pb1—O1	2.566 (8)	C5—C6	1.379 (14)
Pb1—O2	2.609 (8)	C6—C7	1.478 (13)
Pb1—O4	2.851 (8)	C7—C8	1.401 (14)
Pb1—O5	2.675 (9)	C8—C9	1.375 (16)
Pb1—O8	2.693 (8)	C9—C10	1.339 (18)
Pb1—N1	2.618 (8)	C10—C11	1.396 (16)
Pb1—N2	2.528 (8)	C12—C13	1.512 (15)
Pb2—O9	2.624 (7)	C13—C14	1.392 (16)
Pb2—O10	2.763 (11)	C14—C15	1.36 (2)
Pb2—O11	2.629 (8)	C15—C16	1.375 (18)
Pb2—N6	2.470 (7)	C16—C17	1.397 (14)
Pb2—N7	2.422 (8)	C17—C18	1.480 (13)
Pb2—O12^i^	2.910 (9)	C18—C19	1.403 (15)
O1—N3	1.269 (13)	C19—C20	1.389 (18)
O2—N3	1.261 (12)	C20—C21	1.348 (19)
O3—N3	1.222 (12)	C21—C22	1.384 (17)
O4—N4	1.245 (13)	C1—H1A	0.9600
O5—N4	1.287 (13)	C1—H1B	0.9600
O6—N4	1.192 (14)	C1—H1C	0.9600
O7—N5	1.241 (13)	C3—H3	0.9300
O8—N5	1.247 (10)	C4—H4	0.9300
O9—N5	1.275 (12)	C5—H5	0.9300
O10—N8	1.226 (13)	C8—H8	0.9300
O11—N8	1.244 (12)	C9—H9	0.9300
O12—N8	1.244 (12)	C10—H10	0.9300
N1—C2	1.354 (13)	C11—H11	0.9300
N1—C6	1.349 (12)	C12—H12A	0.9600
N2—C7	1.333 (12)	C12—H12B	0.9600
N2—C11	1.312 (14)	C12—H12C	0.9600
N6—C13	1.338 (13)	C14—H14	0.9300
N6—C17	1.350 (12)	C15—H15	0.9300
N7—C18	1.348 (12)	C16—H16	0.9300
N7—C22	1.329 (14)	C19—H19	0.9300
C1—C2	1.477 (15)	C20—H20	0.9300
C2—C3	1.376 (16)	C21—H21	0.9300
C3—C4	1.354 (19)	C22—H22	0.9300
C4—C5	1.365 (18)		
			
O1—Pb1—O2	49.6 (3)	N1—C2—C3	119.6 (10)
O1—Pb1—O4	105.4 (3)	C1—C2—C3	123.3 (10)
O1—Pb1—O5	84.7 (3)	C2—C3—C4	120.6 (12)
O1—Pb1—O8	166.8 (3)	C3—C4—C5	120.0 (12)
O1—Pb1—N1	111.9 (3)	C4—C5—C6	118.8 (11)
O1—Pb1—N2	73.1 (3)	N1—C6—C5	121.1 (9)
O2—Pb1—O4	133.0 (3)	N1—C6—C7	116.2 (8)
O2—Pb1—O5	134.1 (3)	C5—C6—C7	122.7 (9)
O2—Pb1—O8	141.9 (2)	N2—C7—C6	118.4 (8)
O2—Pb1—N1	83.7 (2)	N2—C7—C8	120.7 (9)
O2—Pb1—N2	94.9 (3)	C6—C7—C8	120.8 (8)
O4—Pb1—O5	45.5 (3)	C7—C8—C9	118.9 (10)
O4—Pb1—O8	71.0 (3)	C8—C9—C10	119.8 (10)
O4—Pb1—N1	140.2 (3)	C9—C10—C11	118.6 (11)
O4—Pb1—N2	116.8 (3)	N2—C11—C10	122.6 (10)
O5—Pb1—O8	84.0 (3)	N6—C13—C12	117.1 (9)
O5—Pb1—N1	124.2 (3)	N6—C13—C14	121.8 (10)
O5—Pb1—N2	72.2 (3)	C12—C13—C14	121.1 (10)
O8—Pb1—N1	69.5 (2)	C13—C14—C15	119.5 (12)
O8—Pb1—N2	96.9 (3)	C14—C15—C16	120.0 (12)
N1—Pb1—N2	63.9 (3)	C15—C16—C17	118.2 (12)
O9—Pb2—O10	141.1 (3)	N6—C17—C16	122.1 (9)
O9—Pb2—O11	141.2 (3)	N6—C17—C18	115.9 (7)
O9—Pb2—N6	70.3 (2)	C16—C17—C18	122.0 (9)
O9—Pb2—N7	75.0 (2)	N7—C18—C17	118.5 (8)
O9—Pb2—O12^i^	117.1 (2)	N7—C18—C19	119.6 (9)
O10—Pb2—O11	46.0 (3)	C17—C18—C19	121.9 (9)
O10—Pb2—N6	75.8 (3)	C18—C19—C20	118.3 (11)
O10—Pb2—N7	109.1 (3)	C19—C20—C21	121.0 (11)
O10—Pb2—O12^i^	101.8 (3)	C20—C21—C22	118.3 (12)
O11—Pb2—N6	81.6 (2)	N7—C22—C21	122.0 (11)
O11—Pb2—N7	69.7 (3)	C2—C1—H1A	110.00
O11—Pb2—O12^i^	74.6 (3)	C2—C1—H1B	110.00
N6—Pb2—N7	67.5 (3)	C2—C1—H1C	110.00
O12^i^—Pb2—N6	147.3 (3)	H1A—C1—H1B	109.00
O12^i^—Pb2—N7	83.3 (3)	H1A—C1—H1C	109.00
Pb1—O1—N3	96.4 (6)	H1B—C1—H1C	109.00
Pb1—O2—N3	94.6 (6)	C2—C3—H3	120.00
Pb1—O4—N4	94.8 (6)	C4—C3—H3	120.00
Pb1—O4—Pb1^ii^	110.9 (3)	C3—C4—H4	120.00
Pb1^ii^—O4—N4	153.4 (7)	C5—C4—H4	120.00
Pb1—O5—N4	102.2 (6)	C4—C5—H5	121.00
Pb1—O8—N5	120.0 (6)	C6—C5—H5	121.00
Pb2—O9—N5	110.7 (5)	C7—C8—H8	121.00
Pb2—O10—N8	94.1 (7)	C9—C8—H8	121.00
Pb2—O11—N8	100.3 (6)	C8—C9—H9	120.00
Pb2^i^—O12—N8	106.0 (7)	C10—C9—H9	120.00
Pb1—N1—C2	119.9 (6)	C9—C10—H10	121.00
Pb1—N1—C6	116.9 (6)	C11—C10—H10	121.00
C2—N1—C6	119.9 (8)	N2—C11—H11	119.00
Pb1—N2—C7	120.8 (6)	C10—C11—H11	119.00
Pb1—N2—C11	119.8 (7)	C13—C12—H12A	109.00
C7—N2—C11	119.2 (9)	C13—C12—H12B	109.00
O1—N3—O2	118.2 (8)	C13—C12—H12C	110.00
O1—N3—O3	121.0 (10)	H12A—C12—H12B	109.00
O2—N3—O3	120.7 (10)	H12A—C12—H12C	109.00
O4—N4—O5	115.7 (9)	H12B—C12—H12C	109.00
O4—N4—O6	123.4 (10)	C13—C14—H14	120.00
O5—N4—O6	120.9 (10)	C15—C14—H14	120.00
O7—N5—O8	122.7 (9)	C14—C15—H15	120.00
O7—N5—O9	119.5 (8)	C16—C15—H15	120.00
O8—N5—O9	117.8 (8)	C15—C16—H16	121.00
Pb2—N6—C13	122.8 (6)	C17—C16—H16	121.00
Pb2—N6—C17	118.7 (5)	C18—C19—H19	121.00
C13—N6—C17	118.4 (8)	C20—C19—H19	121.00
Pb2—N7—C18	118.9 (6)	C19—C20—H20	119.00
Pb2—N7—C22	119.8 (7)	C21—C20—H20	119.00
C18—N7—C22	120.7 (9)	C20—C21—H21	121.00
O10—N8—O11	117.3 (9)	C22—C21—H21	121.00
O10—N8—O12	121.4 (10)	N7—C22—H22	119.00
O11—N8—O12	121.3 (9)	C21—C22—H22	119.00
N1—C2—C1	117.2 (9)		
			
O2—Pb1—O1—N3	−6.2 (6)	O11—Pb2—N7—C22	87.4 (8)
O4—Pb1—O1—N3	127.9 (6)	N6—Pb2—N7—C22	176.5 (8)
O5—Pb1—O1—N3	168.9 (7)	O12^i^—Pb2—N7—C22	11.4 (8)
N1—Pb1—O1—N3	−66.3 (7)	O12^i^—Pb2—N6—C13	−155.8 (6)
N2—Pb1—O1—N3	−118.2 (7)	O9—Pb2—N6—C17	−83.2 (6)
O4^ii^—Pb1—O1—N3	54.0 (7)	O10—Pb2—N6—C17	116.3 (6)
O1—Pb1—O2—N3	6.2 (6)	O11—Pb2—N6—C17	69.7 (6)
O4—Pb1—O2—N3	−65.2 (7)	N7—Pb2—N6—C17	−1.8 (6)
O5—Pb1—O2—N3	−0.8 (8)	O12^i^—Pb2—N6—C17	26.5 (8)
O8—Pb1—O2—N3	176.8 (5)	O9—Pb2—N7—C18	80.0 (6)
N1—Pb1—O2—N3	132.1 (6)	O10—Pb2—N7—C18	−59.4 (7)
N2—Pb1—O2—N3	69.1 (6)	O11—Pb2—N7—C18	−83.6 (6)
O4^ii^—Pb1—O2—N3	−122.8 (6)	N6—Pb2—N7—C18	5.5 (6)
O1—Pb1—O4—N4	73.9 (7)	O12^i^—Pb2—N7—C18	−159.6 (6)
O2—Pb1—O4—N4	122.3 (6)	O9—Pb2—N6—C13	94.5 (7)
O5—Pb1—O4—N4	7.7 (6)	O10—Pb2—N6—C13	−66.0 (7)
O8—Pb1—O4—N4	−92.9 (7)	O11—Pb2—N6—C13	−112.6 (7)
N1—Pb1—O4—N4	−85.2 (8)	N7—Pb2—N6—C13	175.9 (7)
N2—Pb1—O4—N4	−4.7 (8)	O9—Pb2—N7—C22	−109.0 (8)
O4^ii^—Pb1—O4—N4	−173.2 (7)	O10—Pb2—N7—C22	111.6 (8)
O1—Pb1—O4—Pb1^ii^	−113.0 (3)	Pb1—O1—N3—O3	−168.1 (10)
O2—Pb1—O4—Pb1^ii^	−64.5 (5)	Pb1—O1—N3—O2	11.1 (10)
O5—Pb1—O4—Pb1^ii^	−179.1 (6)	Pb1—O2—N3—O1	−10.8 (10)
O8—Pb1—O4—Pb1^ii^	80.3 (3)	Pb1—O2—N3—O3	168.4 (10)
N1—Pb1—O4—Pb1^ii^	88.0 (5)	Pb1^ii^—O4—N4—O5	−178.4 (13)
N2—Pb1—O4—Pb1^ii^	168.5 (3)	Pb1—O4—N4—O6	167.5 (11)
O4^ii^—Pb1—O4—Pb1^ii^	0.0 (3)	Pb1—O4—N4—O5	−12.8 (10)
O1^ii^—Pb1^ii^—O4—Pb1	−97.2 (4)	Pb1^ii^—O4—N4—O6	2(2)
O2^ii^—Pb1^ii^—O4—Pb1	−138.7 (4)	Pb1—O5—N4—O4	13.9 (11)
O4^ii^—Pb1^ii^—O4—Pb1	0.0 (3)	Pb1—O5—N4—O6	−166.4 (10)
O5^ii^—Pb1^ii^—O4—Pb1	−0.7 (5)	Pb1—O8—N5—O9	81.2 (9)
O8^ii^—Pb1^ii^—O4—Pb1	75.2 (3)	Pb1—O8—N5—O7	−99.6 (10)
N1^ii^—Pb1^ii^—O4—Pb1	138.6 (3)	Pb2—O9—N5—O8	177.8 (6)
O1^ii^—Pb1^ii^—O4—N4	67.4 (18)	Pb2—O9—N5—O7	−1.4 (11)
O2^ii^—Pb1^ii^—O4—N4	26.0 (17)	Pb2—O10—N8—O11	14.9 (10)
O4^ii^—Pb1^ii^—O4—N4	164.7 (18)	Pb2—O10—N8—O12	−166.7 (9)
O5^ii^—Pb1^ii^—O4—N4	164.0 (17)	Pb2—O11—N8—O10	−15.9 (10)
O8^ii^—Pb1^ii^—O4—N4	−120.2 (17)	Pb2—O11—N8—O12	165.7 (8)
N1^ii^—Pb1^ii^—O4—N4	−56.7 (18)	Pb2^i^—O12—N8—O10	129.4 (9)
O1—Pb1—O5—N4	−125.3 (7)	Pb2^i^—O12—N8—O11	−52.2 (11)
O2—Pb1—O5—N4	−120.0 (7)	Pb1—N1—C2—C1	−20.6 (11)
O4—Pb1—O5—N4	−7.6 (6)	C6—N1—C2—C1	−179.4 (8)
O8—Pb1—O5—N4	61.6 (7)	Pb1—N1—C2—C3	158.7 (7)
N1—Pb1—O5—N4	121.8 (6)	C6—N1—C2—C3	−0.1 (13)
N2—Pb1—O5—N4	160.8 (8)	C2—N1—C6—C5	1.3 (13)
O4^ii^—Pb1—O5—N4	−8.5 (8)	Pb1—N1—C6—C7	22.2 (10)
O2—Pb1—O8—N5	141.4 (7)	C2—N1—C6—C7	−178.4 (8)
O4—Pb1—O8—N5	4.4 (7)	Pb1—N1—C6—C5	−158.2 (7)
O5—Pb1—O8—N5	−40.4 (7)	C11—N2—C7—C6	−178.7 (9)
N1—Pb1—O8—N5	−170.4 (8)	Pb1—N2—C7—C8	179.4 (7)
N2—Pb1—O8—N5	−111.6 (7)	C7—N2—C11—C10	−5.9 (16)
O4^ii^—Pb1—O8—N5	77.1 (7)	Pb1—N2—C11—C10	178.3 (8)
O8—Pb1—N1—C6	92.2 (6)	Pb1—N2—C7—C6	−3.1 (11)
N2—Pb1—N1—C6	−16.8 (6)	C11—N2—C7—C8	3.7 (14)
O4^ii^—Pb1—N1—C6	159.2 (6)	Pb2—N6—C17—C16	178.1 (7)
O1—Pb1—N2—C7	135.5 (7)	C13—N6—C17—C16	0.3 (13)
O2—Pb1—N2—C7	90.4 (7)	Pb2—N6—C13—C14	−178.6 (8)
O4—Pb1—N2—C7	−125.4 (7)	Pb2—N6—C13—C12	0.1 (12)
O5—Pb1—N2—C7	−134.7 (7)	C17—N6—C13—C12	177.7 (8)
O8—Pb1—N2—C7	−53.3 (7)	Pb2—N6—C17—C18	−1.6 (9)
N1—Pb1—N2—C7	9.9 (6)	C17—N6—C13—C14	−0.9 (14)
O1—Pb1—N2—C11	−48.8 (8)	C13—N6—C17—C18	−179.4 (8)
O2—Pb1—N2—C11	−93.9 (8)	Pb2—N7—C22—C21	−172.4 (9)
O4—Pb1—N2—C11	50.2 (8)	Pb2—N7—C18—C19	172.8 (7)
O5—Pb1—N2—C11	40.9 (8)	Pb2—N7—C18—C17	−8.6 (10)
O8—Pb1—N2—C11	122.4 (8)	C18—N7—C22—C21	−1.5 (16)
N1—Pb1—N2—C11	−174.4 (8)	C22—N7—C18—C17	−179.5 (9)
O5—Pb1—N1—C6	25.0 (7)	C22—N7—C18—C19	1.9 (13)
O5—Pb1—N1—C2	−134.5 (7)	C1—C2—C3—C4	177.5 (11)
O8—Pb1—N1—C2	−67.3 (7)	N1—C2—C3—C4	−1.7 (16)
N2—Pb1—N1—C2	−176.2 (8)	C2—C3—C4—C5	2.3 (18)
O4^ii^—Pb1—N1—C2	−0.2 (7)	C3—C4—C5—C6	−1.1 (17)
O1—Pb1—N1—C2	126.8 (7)	C4—C5—C6—N1	−0.7 (14)
O2—Pb1—N1—C2	85.1 (7)	C4—C5—C6—C7	179.0 (9)
O4—Pb1—N1—C2	−75.0 (8)	N1—C6—C7—C8	164.3 (9)
O4—Pb1—N1—C6	84.5 (7)	N1—C6—C7—N2	−13.3 (12)
O1—Pb1—N1—C6	−73.7 (7)	C5—C6—C7—C8	−15.4 (14)
O2—Pb1—N1—C6	−115.4 (6)	C5—C6—C7—N2	167.0 (9)
O10^i^—Pb2^i^—O12—N8	11.7 (7)	C6—C7—C8—C9	−178.9 (10)
O11^i^—Pb2^i^—O12—N8	−25.8 (7)	N2—C7—C8—C9	−1.3 (15)
N6^i^—Pb2^i^—O12—N8	−70.4 (8)	C7—C8—C9—C10	1.2 (17)
N7^i^—Pb2^i^—O12—N8	−96.6 (7)	C8—C9—C10—C11	−3.2 (18)
O9^i^—Pb2^i^—O12—N8	−165.9 (6)	C9—C10—C11—N2	5.8 (18)
O10—Pb2—O9—N5	−74.9 (7)	C12—C13—C14—C15	−178.1 (11)
O11—Pb2—O9—N5	−151.9 (6)	N6—C13—C14—C15	0.5 (16)
N6—Pb2—O9—N5	−105.8 (6)	C13—C14—C15—C16	0.6 (18)
N7—Pb2—O9—N5	−176.8 (7)	C14—C15—C16—C17	−1.2 (17)
O12^i^—Pb2—O9—N5	109.0 (6)	C15—C16—C17—N6	0.8 (15)
O9—Pb2—O10—N8	−130.6 (6)	C15—C16—C17—C18	−179.6 (9)
O11—Pb2—O10—N8	−8.6 (6)	C16—C17—C18—C19	5.6 (14)
N6—Pb2—O10—N8	−100.6 (7)	N6—C17—C18—C19	−174.7 (8)
N7—Pb2—O10—N8	−41.0 (7)	C16—C17—C18—N7	−173.0 (8)
O12^i^—Pb2—O10—N8	45.9 (7)	N6—C17—C18—N7	6.7 (11)
O9—Pb2—O11—N8	130.3 (6)	C17—C18—C19—C20	179.8 (10)
O10—Pb2—O11—N8	8.6 (6)	N7—C18—C19—C20	−1.7 (15)
N6—Pb2—O11—N8	87.0 (6)	C18—C19—C20—C21	1.1 (18)
N7—Pb2—O11—N8	156.0 (7)	C19—C20—C21—C22	−0.7 (19)
O12^i^—Pb2—O11—N8	−115.5 (6)	C20—C21—C22—N7	0.9 (19)

Symmetry codes: (i) −*x*+1, −*y*, −*z*; (ii) −*x*+2, −*y*+1, −*z*+1.

### Hydrogen-bond geometry (Å, °)

**Table:**

*D*—H···*A*	*D*—H	H···*A*	*D*···*A*	*D*—H···*A*
C1—H1A···O4^ii^	0.96	2.55	3.378 (14)	144
C3—H3···O10^iii^	0.93	2.50	3.368 (17)	155
C10—H10···O6^iv^	0.93	2.47	3.349 (17)	157
C12—H12A···O10	0.96	2.48	3.299 (15)	143
C12—H12B···O7	0.96	2.49	3.412 (17)	160
C12—H12C···O8^iii^	0.96	2.59	3.516 (14)	162
C19—H19···O9^v^	0.93	2.45	3.253 (13)	145
C21—H21···O1^iv^	0.93	2.42	3.212 (15)	143
C22—H22···O12^i^	0.93	2.53	3.268 (15)	137

Symmetry codes: (ii) −*x*+2, −*y*+1, −*z*+1; (iii) −*x*+1, −*y*+1, −*z*+1; (iv) −*x*+2, −*y*, −*z*+1; (v) −*x*+1, −*y*, −*z*+1; (i) −*x*+1, −*y*, −*z*.
